# Sexual behaviors: study in the youth

**DOI:** 10.1590/S1679-45082018AO4265

**Published:** 2018-09-17

**Authors:** Patrícia Sofia Ferreira Miranda, Joana Margarida Gonçalves Aquino, Ricardo Miguel Patrício de Carvalho Monteiro, Maria dos Anjos Coelho Rodrigues Dixe, Alexandra Maria Branco da Luz, Pascoal Moleiro

**Affiliations:** 1 Centro Hospitalar de Leiria, Leiria, Portugal; 2 Centro de Inovação em Tecnologias e Cuidados de Saúde, Instituto Politécnico de Leiria, Leiria, Portugal

**Keywords:** Sexual behavior, Adolescent, Contraception, Sexually transmitted diseases, Pregnancy, Comportamento sexual, Adolescente, Anticoncepção, Doenças sexualmente transmissíveis, Gravidez

## Abstract

**Objective:**

To characterize sexual behaviors in a sample of adolescents and youth.

**Methods:**

An analytical descriptive study using a questionnaire about sexual behaviors, adapted from the World Health Organization. It was distributed to students from a Portuguese city aged 14-24 years, during two months. Two age groups were defined: G1 - students aged 14-19 years; G2 - aged 20-24 years.

**Results:**

The sample included 2,369 students, 61% females and 70% in G1. The mean age of first sexual intercourse was 16.4±1.8 years; 93% used some contraceptive method in the first sexual intercourse. Out of those who did not use contraception in the first sexual intercourse, 83% were in G1 (p<0.001). Emergency contraception was used at least once by 54% (63% in G2, p<0.001). Among those who had unprotected sexual intercourses, 9% were under the influence of alcohol, 53.6% were female and 53.4% were in G2 (p<0.001). Homosexual contacts occurred in 21% of cases; in that, 62% in G1 and 84% among females (p<0.001).

**Conclusion:**

The use of contraceptionin the first sexual intercourse was common in our sample. However, the number of adolescents not using any contraceptive method in subsequent sexual intercourses, and the high percentage of them who consider it unnecessary, are a concern. Unprotected sexual intercourses, as well as unplanned intercourses and under influence of alcohol or drugs, especially in the youngest, urge the need for intervention regarding sexual education.

## RESUMO

**Objetivo:**

Caracterizar os comportamentos sexuais em uma amostra de adolescentes e jovens.

**Métodos:**

Estudo descritivo analítico, utilizando um questionário sobre comportamentos sexuais adaptado da Organização Mundial da Saúde. O questionário foi distribuído a estudantes entre os 14 e os 24 anos de uma cidade portuguesa, em período de 2 meses. Definiram-se dois grupos etários: G1, para estudantes de 14 a 19 anos; e G2, de 20 a 24 anos.

**Resultados:**

Da amostra, constaram 2.369 estudantes, sendo 61% do sexo feminino e 70% do G1. A média de idade da primeira relação sexual foi de 16,4± 1,8 anos. Foi utilizado método contraceptivo na primeira relação sexual em 93%. Dos que não utilizaram, 83% pertenciam ao G1 (p<0,001). Entre os estudantes, 54% recorreram pelo menos uma vez à contracepção de emergência (63% do G2; p<0,001). Dos que tiveram relações sexuais desprotegidas, 9% estavam sob influência do álcool. Destes últimos, 53,6% eram do sexo feminino e 53,4% pertenciam ao G2 (p<0,001). Os contatos homossexuais ocorreram em 21% dos casos, dos quais 62% eram do G1 e 84% do sexo feminino (p<0,001).

**Conclusão:**

O recurso à contracepção na primeira relação sexual foi uma prática comum na nossa amostra. No entanto, o número de adolescentes que não utiliza método contraceptivo nas relações subsequentes e a elevada percentagem que o considera desnecessária são preocupantes. Para além das relações sexuais desprotegidas, as relações não planejadas e sob o efeito de álcool ou drogas, sobretudo entre os mais novos, reforçam a necessidade de intervenção no âmbito da educação sexual.

## INTRODUCTION

The World Health Organization (WHO) defines adolescence as the period between 10 and 19 years of age,^(^ ^1^ ^)^ and youth as between 15 and 24 years.^(^ ^1^ ^,^ ^2^ ^)^

The causes of morbidity and mortality in adolescence and youth have been modified in the last decades, with an increase in causes resulting from lifestyles that could be prevented.^(^ ^3^ ^)^ Pregnancy in adolescence and sexually transmitted diseases (STDs) are currently the primary causes of morbidity.^(^ ^4^ ^)^

Adolescents and youth build their identity by integration of feelings and desires,^(^ ^3^ ^,^ ^4^ ^)^ and sexual activity often begins in this period. This change is not always accompanied by appropriate sexual education or by knowledge about physiology or biological aspects of sexuality and reproduction.^(^ ^4^ ^)^

Several studies revealed that adolescents initiate their sexual activity increasingly earlier.^(^ ^3^ ^,^ ^5^ ^)^ This seems to be associated with the existence of multiple sexual partners, high rates of STDs, emotional disorders, greater precociousness in consuming alcohol, tobacco, and drugs, higher rates of abortion, complications during pregnancy, and preterm births.^(^ ^6^ ^)^

Despite observing an improvement in the sexual behaviors of adolescents and youth in the last years, contraception is not always a priority at the start of sexual life.^(^ ^3^ ^)^ Many do not use any type of contraceptive, or use the condom incorrectly, which increases the risk of undesired pregnancy and STDs.^(^ ^3^ ^,^ ^4^ ^)^

Sexual education and health promotion should take place before sexual activity is initiated.^(^ ^7^ ^)^ The healthcare professional plays a fundamental role in promotion of reproductive health and in contraceptive orientation,^(^ ^8^ ^)^ and it is equally important to involve the parents in the dialogue about sexuality with their children.^(^ ^9^ ^)^

The good practices in preventive care given to adolescents and youth include the drafting of their sexual and reproductive history, tracking pregnancy and STDs, as well as counseling and access to contraceptive methods.^(^ ^3^ ^)^

Prevention of pregnancy and STDs in this age range is a central theme in 21^st^ century health care, as they are significant causes of health, social, and economic problems of adolescents and youth, as well as of society in general.^(^ ^3^ ^)^

Therefore, there is great importance in knowing the sexual behaviors of adolescents and youth, bearing in mind early intervention. The primary objective of the study is to assess knowledge and characterize the sexual behaviors of adolescents and youth of Leiria, a city in Portugual. The secondary objective is to compare them with national and international data, seeking to implement measures that allow decreasing morbidity resulting from sexual risk behaviors.

## OBJECTIVE

To characterize the sexual behaviors and knowledge about sexuality of adolescents and youth, as well as to evaluate risk behaviors and identify the respective protection and risk factors.

## METHODS

This is an analytical descriptive study, based on a questionnaire adapted from the original developed by the WHO, comprising multiple-choice questions, in order to evaluate knowledge and sexual behaviors of adolescents. It is a questionnaire containing questions related to the use of contraceptive methods, sexual practices, STDs, pregnancy, emergency contraception, concomitant consumptions, and sexual orientation. The original questionnaire is available from http://www.who.int/reproductivehealth/topics/adolescence/questionnaire/en/

The questionnaire was distributed to Portuguese students of Secondary and Higher Education, enrolled, respectively, at secondary schools of the county of Leiria and at the *Instituto Politécnico de Leiria,* aged between 14 and 24 years. This was an individual, anonymous, confidential, and voluntary questionnaire, to be completed in the classroom during approximately 20 minutes. The questionnaire was distributed by teachers to groups of 20 to 30 students of secondary school and later collected, in sealed envelopes, by healthcare professionals, responsible for the study. In the higher education cases, the questionnaire was present on an online platform, equally guaranteeing anonymity and confidentiality. Data were collected during the period between April and May 2013.

The sample was divided into two age groups: Group 1 (G1), including those aged 14-19 years, and Group 2 (G2), from 20 to 24 years. The sample was analyzed per sex and age group.

The variable studies were first sexual intercourse, sexual behaviors, contraceptive methods, pregnancy, and STDs.

For statistical analysis, the Statistical Package for Social Science (SPSS), version 22 (α=0.05) was used. The χ^2^ test was utilized to compare categorical variables.

The project was approved by the General Administration of Education (protocol no. 0366900002) and by the deans of the schools. In order to respect ethical principles, informed consent was previously obtained from the parents of the students under 18 years, and from the students themselves for participation in the study.

## RESULTS

This investigation involved 2,369 adolescents and youth, distributed by age and by sex, as shown in table 1. The mean age was 18.5±2.4 years, and the median was 18 years.

**Table 1:** Characterization of the sample

Groups	Males	Females
	n (%)	n (%)
1	601 (38.3)	970 (61.7)
2	266 (38.8)	420(61.2)

Fifty-eight percent of adolescents and youth of the sample had already initiated sexual life; in that, 66% were female (p=0.993) and 74.5% from G2 (p<0.001).

The mean age at the first sexual intercourse was 16.4±1.8 years (16.4±1.7 years for females and 16.4±1.9 for males), with a statistically significant difference between the age groups: 15.9±1.3 years in G1, and 17±1.9 years in G2 (p<0.001).

The first sexual intercourse occurred with the boyfriend/girlfriend in 78.5% of cases, and in 88%, it was consensual. Three and a half per cent (75% females; p=0.009) admitted they were forced to have sexual intercourse, 2% had sex intercourse in exchange for money (68% males; p<0.001), and 13% admitted to having several partners simultaneously.

Participants admitted they had already adopted other forms of sexual behaviors, especially practices of self-masturbation (63%) and heteromasturbation (56%), and 30% admitted to have watched pornographic films. As to type of sex, 59% had already engaged in vaginal sex; 53% admitted to having had oral sex; 23% anal sex; and 3% had already participated in group sex.

Relative to those who referred not having initiated a sex life, the mean age was 19.9±1.6 years, in which 66% were females (p=0.993) and 51% were from G2 (p<0.001). The reasons that motivated postponing initiation of sexual activity are displayed in table 2. Of these, 74% admitted to having someone to whom they had already felt attracted sexually or sentimentally. Others admitted that they had already adopted other sexual behavior, such as self-masturbation (63%), watching pornographic films (29%), heteromasturbation (27%), oral sex (22.5%), and anal sex (6%).

**Table 2:** Reasons for postponing initiation of sexual life and sexual orientation

	n	Male	Female	p value	Group 1	Group 2	p value
	(%)	n (%)	n (%)		n (%)	n (%)	
I feel I'm not prepared	345(43)	42 (12.2)	303 (87.8)	<0.001	323 (99.7)	1 (0.3)	0.005
I have not had an opportunity	472 (51.9)	195 (41.3)	277 (58.7)	0.013	431 (46.2)	17(3.8)	<0.001
I believe we must have no sexual intercourse before getting married	120 (13.2)	45 (37.5)	75 (62.5)	0.035	74 (64.9)	40(35.1)	<0.001
I fear making someone pregnant / getting pregnant	281(31.5)	77 (27.4)	204 (72.6)	<0.001	264 (97.8)	6 (2.2)	<0.001
I am afraid of acquiring HIV or other STI	387(43.6)	118 (30.5)	269 (69.5)	0.001	361 (96.5)	13 (3.5)	<0.001
I believe I am very young	336 (36.8)	74 (22)	262 (78)	<0.001	302 (95.3)	15 (4.7)	<0.001
I fear being disliked by society	66 (7.3)	23 (34.8)	43 (65.2)	0.818	61 (98.4)	1 (1.6)	0.112
Opposite sex	1,970 (91.4)	809 (41.1)	1,160 (58.9)	0.001	1,305 (68.9)	588(31.1)	0.002
Same sex	73 (3.4)	15 (20.5)	58 (79.5)		36 (50)	36 (50)	
Both sexes	112(5.2)	37 (33)	75 (67)		81 (73)	30 (27)	

STI: Sexually transmitted infections

Twenty-one percent of adolescents and youth referred having had homosexual experiences, in which 62% belonged to G1 and 84% were female (p<0.001). Sexual orientation is detailed on table 2.

In the first sexual relation, a contraceptive method was used by 93% of adolescents and youth, 60% of whom were female (p<0.001), and 54% from G1 (p=0.499).

The first sexual intercourse was considered accidental by 59% of female adolescents and youth (p=0.402), and by 55% of G1 (p=0.147). Among those who admitted having had an accidental first sexual intercourse (51%), 91% used some contraceptive method (p< 0.001). On the other hand, of those who had planned sexual relations, 96% used contraception (p<0.001).

The contraceptive of choice in the first sexual intercourse was condom (85%), while in subsequent sexual intercourses, the most frequently used methods were condom (34%) and dual protection (35%). The use of contraception and the contraceptive methods chosen are detailed on tables 3 and 4, respectively.

**Table 3:** Use of contraception and consumption of alcohol/drugs

	Male	Female	p value	Group 1	Group 2	p value
	n (%)	n (%)		n (%)	n (%)	
First sexual intercourse	514 (39.7)	780(60.3)	0.014	683 (54.3)	574 (45.7)	0.915
Subsequent sexual intercourses	504 (37.2)	852(62.8)	0.651	729 (55.1)	595 (44.9)	<0.001
Consumption of alcohol	274 (46.4)	317 (53.6)	<0.001	270 (46.6)	310 (53.4)	<0.001
Consumption of other drugs	102 (47.2)	114 (52.8)	0.003	106 (51)	102(49)	<0.001

**Table 4:** Contraceptive methods chosen in the first and subsequent sexual intercourses

Contraceptive methods	First intercourse n (%)	Subsequent intercourses n (%)
Condom	1119 (85.1)	471 (33.6)
Pill	95 (7.2)	323 (23)
*Coitus interruptus*	22 (1.7)	5 (0.4)
Pill + condom (dual protection)	73(5.6)	496 (35.4)
Calendar	4 (0.3)	-
Pill + *coitus interruptus*	2 (0.2)	23 (1.6)
Pill + *coitus interruptus*	-	19 (1.4)
Condom + pill + *coitus interruptus*	-	56 (4)

Among the participants who did not use contraception after the first sexual intercourse (18%), 83.5% belonged to G1 (p<0.001), and 61% were female (p=0.651). Of these, 70% considered contraception unnecessary (73% females, and 69% of G2; p<0.001).

In our sample, 54% of adolescents and youth sought emergency contraception at least once, and of these, 62.5% were female (p=0.404), and 61% belonged to G2 (p<0.001).

At the first sexual intercourse, 26 adolescents/youth (2%) became pregnant, and 24 of these pregnancies (77%) ended in abortion. From the total sample, 45 adolescents/youth (2%) referred having contracted some sexually transmitted infection (STI).

Among those who had unprotected sexual intercourse, 9% were under the effect of alcohol, and 5% under the influence of other drugs (the distribution per sex and age group is detailed on table 3). Of those who had sexual relations under the influence of other drugs, 53% were female (p=0.002), and 51% belonged to G1 (p<0.001).

## DISCUSSION

Studies developed in several countries revealed that adolescents and youth initiate their sexual activity at earlier age,^(^ ^3^ ^-^ ^5^ ^,^ ^10^ ^)^ and Portugal is not an exception.^(^ ^4^ ^)^ Approximately 40% of youth aged between 13 and 21 years are sexually active.^(^ ^11^ ^)^ According to the report of the Centers for Disease Control and Prevention, 44.1% of adolescent population stated being sexually active.^(^ ^12^ ^)^ In this present study, more than half of those involved (58%) had already initiated their sexual activity, and the mean age at the first sexual intercourse was 16.4 years.

Female adolescents, in general, initiate their sexual life later.^(^ ^3^ ^,^ ^4^ ^,^ ^13^ ^-^ ^16^ ^)^ In this study, the age of initiation was similar in both sexes, which is in accordance with some recent publications.(^11^ ^)^ However, a statistically significance difference was observed among the age groups - younger adolescents initiated their sexual activity earlier (mean 15.9 years), comparatively to the group of older adolescents and youth (mean 17 years).

In the present study, the mean age at the first sexual intercourse was slightly higher than that of other national publications. In a systematic review of sexuality of Portuguese adolescents, the mean age at the first intercourse was 15.6 years.^(^ ^4^ ^)^ The study Health Behavior in School-aged Children (HBSC), more than three fourths of the adolescents began their sex life at 14 years or more.^(^ ^17^ ^)^ In other national studies, the mean was 15.49 years^(^ ^3^ ^)^ and 14.68 years,^(^ ^12^ ^)^ which is corroborated by international data (mean of 14 to 16 years).^(^ ^18^ ^)^

The present study demonstrates a certain degree of lack of knowledge on the part of the participants as to the definition of sexual activity, since part of the adolescents and youth who reported not having initiated their sex life, had the practice of self-and heteromasturbation, watching pornographic films, and the practice of oral and anal sex. Since these practices are not perceived as sexual behaviors, the inherent risk is not duly valued, and it is up to healthcare professionals to intervene in education for their prevention.

It is known that the use of contraception has increased.^(^ ^3^ ^)^ In our sample, the majority (93%) referred having used some contraceptive method at the first sexual intercourse, especially females and in the group of the youngest members. This result was superior to that observed in other national studies, in which the use of contraceptives at the first intercourse ranged from 84 to 91%.^(^ ^4^ ^,^ ^16^ ^)^ In subsequent sexual intercourses, this use decreased (82%), while being equally superior in females sex and in the group of the youngest. Among those who did not use contraception in their subsequent sexual intercourses, about half considered it unnecessary, especially older adolescents and females. This might suggest that adolescents and youth of our sample were not duly instructed as to contraception and the risks resulting from inadequate sexual conducts.

Portugal is one of the European Union countries with the lowest rate of sexual education at schools (48%),^(^ ^4^ ^)^ and counseling with healthcare professionals is also not a common practice. A systematic review showed that only one-third of Portuguese adolescents (34%) already had sought healthcare units seeking orientation on contraception or STDs, especially females and with a higher level of schooling.^(^ ^4^ ^)^ Other studies revealed, equally, that more than two thirds of the sexually active adolescents had never attended family planning consultations.^(^ ^3^ ^,^ ^12^ ^)^

The number of individuals presenting some form of STDs was low, similar to the national mean.^(^ ^4^ ^)^ This result can be a consequence of the high percentage of use of the condom and/or of the existence of undiagnosed or asymptomatic infections.^(^ ^3^ ^,^ ^4^ ^)^

National studies demonstrated the condom as the contraceptive method of choice in the first intercourse (76 to 96%;^(^ ^4^ ^)^ 70.5% as well as in subsequent relations (52 to 69%;^(^ ^4^ ^)^ 70.4%^(^ ^17^ ^)^).

In this study population, the method most used at the first sexual intercourse was the condom, which may be related to its availability and accessibility, to the fact of not requiring a medical prescription, and for providing a low-cost protection against STIs.^(^ ^8^ ^)^

In subsequent sexual relations, the contraceptive methods of choice were condom and dual protection. Less frequent use of condoms in subsequent relations can be justified by immaturity/impulsiveness of adolescents and youth, due to a more advanced age, owing to the use of another contraceptive method by the female partner (in the case of heterosexuals), or because of the existence of a more solid relationship between the couple.

The involvement of youth in more prolonged relationships can represent a barrier to promotion of healthy and safe sexual behaviors.^(^ ^16^ ^)^ This condition may justify the fact of contraception being used less for these older adolescents, as observed in this study. Effectively, the adolescents and youth tend to discontinue the use of the condom as they assume a fixed partner, with whom they have regular sexual activity, especially if they use another method with the intention of preventing pregnancy.^(^ ^19^ ^)^

In 2012, the publications on contraceptive counseling were published by the *Sociedade Portuguesa de Medicina do Adolescente* and the *Sociedade Portuguesa de Contracepção*.^(^ ^20^ ^)^ In these recommendations, dual protection, which associates condom to another highly efficacious method, is the contraception of choice,^(^ ^20^ ^)^ since besides preventing a possible pregnancy, it also prevents STDs. When compared to other international studies, the percentage of use of this method was superior in this sample (35% *versus* <25%).^(^ ^19^ ^)^

In a recent study, 35% of adolescents and youth resorted to emergency contraception at least once. Of these, about half did not use a contraceptive method, and in the others, the method used failed.^(^ ^4^ ^)^

In this study, more than half resorted, at least once, to emergency contraception, especially female adolescents, and almost two thirds belonged to the group of the older youth, which can be related to less use of contraception that was verified in this age group. Greater social exposure of women in the case of pregnancy, and the belief that contraception and the consequences of a pregnancy are the responsibility of females,^(^ ^21^ ^)^ justify the greater search for emergency contraception by women, as well as the greater contraceptive responsibility.

Sexual activity in adolescence is frequently associated with other risk behaviors, especially the consumption of alcohol, tobacco, or other drugs.^(^ ^3^ ^,^ ^10^ ^)^ Alcohol is the most consumed substance, followed by *cannabis.* ^(^ ^22^ ^)^

A large part of the adolescents and youth who already initiated their sex life consumes alcohol regularly,^(^ ^4^ ^)^ and according to some studies, a significant percentage (9%) referred not having used a condom because they were too drunk to use it.^(^ ^4^ ^,^ ^13^ ^)^

Some drugs act as sexual stimulants, reducing inhibition and increasing sexual desire. Those who consume alcohol and other drugs have sexual relations more frequently, are earlier as to initiation of their sex life, have more sex partners, and more unprotected sexual intercourses.^(^ ^22^ ^)^

According to the HBSC study, 15.9% of adolescents have already had sexual intercourses under the effect of alcohol or drugs (19% males and 10.3% females).^(^ ^17^ ^)^ Other national studies indicated that 35 to 51% of adolescents have already had intercourse under the influence of alcohol, and about 22%, under the effect of drugs, predominantly males.^(^ ^22^ ^,^ ^23^ ^)^

In the present study, among the participants who had unprotected sexual intercourses, 9% had previously consumed alcohol and 5% were under the influence of other drugs. Contrary to other studies, in this sample, females had more sexual intercourses under the effect of these substances. As to age, older adolescents had more sexual intercourses under the effect of alcohol, and the younger ones had more intercourses under the influence of drugs, which could be related to the consumption of “light drugs”, which are currently more accessible.

It has been noted that younger adolescents are more involved in unplanned sexual intercourses, which increases the risk of unprotected intercourse and pregnancy.^(^ ^6^ ^)^ Worldwide, 9% of women had an unplanned pregnancy before 19 years of age,^(^ ^15^ ^)^ and in Portugal, this percentage is reduced to 5%, of which 2% between the ages of 15 and 19 years.^(^ ^15^ ^)^ According to the literature, in this study, the younger adolescents and females had more unplanned sexual intercourses.

In Portugal, the birth rate in adolescence is 14.7 births per 1,000 adolescents aged 15 to 19 years, and the abortion rate has decreased.^(^ ^4^ ^)^ In the present study, 2% of adolescents became pregnant, and 77% of these pregnancies resulted in abortions, and the values were similar to those of another national study.^(^ ^3^ ^)^

The reduced number of pregnancies could be related to the high percentage of contraception use. On the other hand, the increase in schooling, the professional perspective, the absence of future plans centered exclusively on maternity, and the ease of access to reproductive health may justify the lowered pregnancy rate in adolescence in Portugal.^(^ ^4^ ^)^ The high number of abortions is likely a result of an unplanned or undesired pregnancy.^(^ ^3^ ^)^

During adolescence, several sexual experiences arise, including homosexual encounters. Homosexual adolescents are a priority group for intervention in healthcare, since they are at a greater risk of social isolation, school failure, family dysfunction, consumption of alcohol or drugs, depression, suicide, and stigmatization.^(^ ^9^ ^)^ Most adolescents in this study showed a preference for heterosexual intercourses, which is in concordance with another Portuguese study.^(^ ^15^ ^)^ The female adolescents referred more frequently to an attraction to the same sex, as was noted in the group of younger adolescents.

Despite the statistical concordance with other national and international studies, this project was limited to one Portuguese city, and may not linearly translate the reality of the country. Nevertheless, the central objective characterized the sexual behaviors of a sample of adolescents/youth, with the intention of outlining preventive and health promoting strategies at the local level, which was verified with the promotion of a forum destined to the adolescents involved (available from: http://www.midlandcom.pt/main.php?link=1132&op=detalhe). In this forum, entitled *Sexualidades à Conversa,* the results of this study were presented and commented on, with a perspective of education and promotion for a healthy and responsible sexuality.

## CONCLUSION

The percentage of contraceptive method use at the first sexual intecourse was high, as well as resorting to dual protection, in agreement with national recommendations. However, the number of adolescents who did not use any contraceptive method in subsequent intercourses, and the high percentage of those who considered it unnecessary were concerning observations.

Beyond unprotected sexual relations, concomitant consumption of alcohol or drugs is a reality that merits special attention. The female adolescents of this study had more encounters under the effect of these substances, as well as more unplanned intercourses, and greatest access to emergency contraception. Added to this is the fact that it was the youngest of them who had the most unplanned relations, sometimes, for lack of access to any contraceptive method and under the effect of alcohol or drugs.

The low rate of pregnancy contrasts with the high percentage of abortion and of emergency contraception use, which in part, might justify this result. Despite the low rate of Sexually Transmitted Infections and of pregnancy, both causes of morbidity in this age range, it is important to invest on implementing preventive and health promoting strategies in order to improve these results. Further, it is worth mentioning that certain practices are not perceived as sexual behaviors, and it is the healthcare professionals’ responsibility to clarify and advise regarding the risk inherent to these practices.

Knowledge of the issues related to sexuality is not yet satisfactory, and are translated by sexual behaviors of risk, such as unprotected sexual intercourses, concomitant consumption of alcohol/drugs, and missing family planning consultations.

Fostering sexual education in Portugal is imperative, involving not only healthcare, but schools and the society in general as well, in order to avoid the consequences from inappropriate sexual conducts.
